# Humoral and Cellular Response Induced by Primary Series and Booster Doses of mRNA Coronavirus Disease 2019 Vaccine in Patients with Cardiovascular Disease: A Longitudinal Study

**DOI:** 10.3390/vaccines12070786

**Published:** 2024-07-17

**Authors:** Yuya Ishihara, Hiroyuki Naruse, Hidetsugu Fujigaki, Reiko Murakami, Tatsuya Ando, Kouhei Sakurai, Komei Uehara, Koki Shimomae, Eirin Sakaguchi, Hidekazu Hattori, Masayoshi Sarai, Junnichi Ishii, Ryosuke Fujii, Hiroyasu Ito, Kuniaki Saito, Hideo Izawa

**Affiliations:** 1 Department of Clinical Laboratory, Fujita Health University Hospital, Toyoake 470-1192, Japan; yuya2105@fujita-hu.ac.jp; 2 Department of Clinical Pathophysiology, Fujita Health University Graduate School of Health Sciences, Toyoake 470-1192, Japan; sakaguch@fujita-hu.ac.jp (E.S.); hhattori@fujita-hu.ac.jp (H.H.); 3 Department of Advanced Diagnostic System Development, Fujita Health University Graduate School of Health Sciences, Toyoake 470-1192, Japan; fujigaki@fujita-hu.ac.jp (H.F.); saitok@fujita-hu.ac.jp (K.S.); 4 Institute for Glyco-Core Research, Gifu University, Yanagido, Gifu 501-1193, Japan; muratani@gifu-u.ac.jp; 5 Department of Joint Research Laboratory of Clinical Medicine, Fujita Health University School of Medicine, Toyoake 470-1192, Japan; andotatstuya@yahoo.co.jp (T.A.); kouhei.sakurai@fujita-hu.ac.jp (K.S.); hiroyasu.ito@fujita-hu.ac.jp (H.I.); 6 Department of Preventive Medical Sciences, Fujita Health University Graduate of Health Sciences, Toyoake 470-1192, Japan; 82023104@fujita-hu.ac.jp (K.U.); 82023111@fujita-hu.ac.jp (K.S.); 7 Department of Cardiology, Fujita Health University School of Medicine, Toyoake 470-1192, Japan; msarai@fujita-hu.ac.jp (M.S.); jishii@fujita-hu.ac.jp (J.I.); izawa@fujita-hu.ac.jp (H.I.); 8 Department of Medical Sciences, Fujita Health University School of Medicine, Toyoake 470-1192, Japan; rfujii@fujita-hu.ac.jp

**Keywords:** mRNA COVID-19 vaccine, cardiovascular disease, humoral and cellular response, longitudinal study

## Abstract

Preexisting cardiovascular disease (CVD) is a pivotal risk factor for severe coronavirus disease 2019 (COVID-19). We investigated the longitudinal (over 1 year and 9 months) humoral and cellular responses to primary series and booster doses of mRNA COVID-19 vaccines in patients with CVD. Twenty-six patients with CVD who received monovalent mRNA COVID-19 vaccines were enrolled in this study. Peripheral blood samples were serially drawn nine times from each patient. IgG against the severe acute respiratory syndrome coronavirus 2 (SARS-CoV-2) spike receptor-binding domain (RBD) was measured using an enzyme-linked immunosorbent assay. The numbers of interferon-γ-releasing cells in response to SARS-CoV-2 peptides were measured using an enzyme-linked immunospot assay. The RBD-IgG titers increased 2 weeks after the primary series and booster vaccination and waned 6 months after vaccination. The S1-specific T cell responses in patients aged < 75 years were favorable before and after booster doses; however, the Omicron BA.1-specific T cell responses were poor. These results suggest that regular vaccination is useful to maintain long-term antibody levels and has implications for booster dose strategies in patients with CVD. Additional booster doses, including Omicron variant-adapted mRNA vaccines, may be recommended for patients with CVD, regardless of age.

## 1. Introduction

Preexisting cardiovascular disease (CVD) is a pivotal risk factor for the severe clinical course of coronavirus disease 2019 (COVID-19) and is linked to unfavorable outcomes [1,2,3,4,5,6,7,8,9]. Furthermore, COVID-19 may worsen underlying heart disease and is frequently associated with cardiovascular complications such as thromboembolic events, severe ventricular arrhythmia, and myocardial injury [10,11]. COVID-19 has been involved in direct damage to the cardiovascular system [12].

Despite advanced made in therapy against COVID-19, vaccination remains the most effective intervention in reducing the morbidity and mortality of this disease [13,14]. The primary series of the COVID-19 vaccines has shown excellent efficacy in clinical trials [15,16] and effectiveness in real-world settings [17]. However, breakthrough COVID-19 infections after vaccination, which are likely due to a combination of diminishing immunity and the emergence of severe acute respiratory syndrome coronavirus 2 (SARS-CoV-2) variants (i.e., Omicron) that can escape vaccine-induced immune response, have led to the need for third and fourth doses of the vaccines, known as boosters [18,19,20,21]. Immune memory to SARS-CoV-2 is induced by booster vaccinations with either BNT162b2 or mRNA-1273 mRNA vaccines, which provide protection against severe COVID-19 by utilizing both cellular and humoral immunity [22]. However, few reports are available on the longitudinal immunological effects of the primary series and booster doses of the mRNA COVID-19 vaccine in patients with CVD.

In the present study, we aimed to evaluate the humoral and cellular responses to primary series and booster doses of the mRNA COVID-19 vaccine over 1 year and 9 months in patients with CVD.

## 2. Materials and Methods

### 2.1. Ethics Statements

The Ethics Committee of Fujita Health University approved this study (protocol number: HM21-392), and it was conducted in accordance with the Declaration of Helsinki. All patients signed an informed consent statement prior to participation in the study.

### 2.2. Participants and Blood Samples

This prospective study was carried out at the Department of Clinical Pathophysiology, Fujita Health University Graduate School of Health Sciences, in cooperation with the Department of Cardiology, Fujita Health University School of Medicine. Between February 2021 and September 2022, patients with CVD who received the monovalent BNT162b2 mRNA COVID-19 vaccine (Pfizer–BioNTech, New York, NY, USA) or the mRNA-1273 mRNA vaccine (Moderna, Cambridge, MA, USA) were enrolled in this study. Patients received BNT162b2 only for their first and second doses (V1 and V2) and either BNT162b2 or mRNA-1273 for their third and fourth doses (V3 and V4). Patients with the following characteristics were excluded from the study: (1) possible current SARS-CoV-2 infection at study enrollment (those with fever and/or respiratory symptoms), (2) a history of COVID-19, (3) clinical or electrocardiographic evidence indicative of acute coronary syndrome or coronary revascularization in the previous 6 months, (4) symptomatic heart failure, (5) malignancy actively managed with chemotherapy or radiotherapy, and (6) any autoimmune diseases.

**Figure 1:**
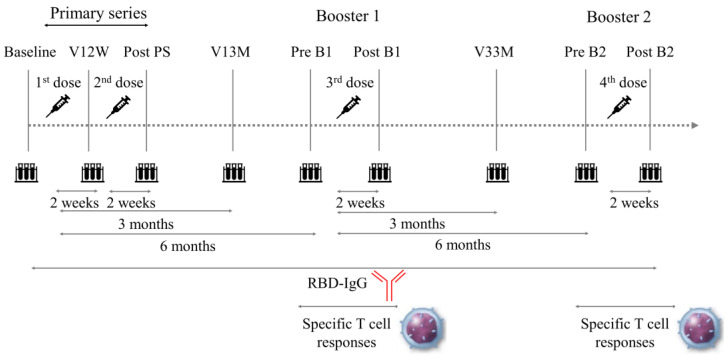
Study timeline. A total of 9 blood samples were collected from 26 outpatients with cardiovascular disease at the following timepoints: including baseline (just before vaccination), V12W (2 weeks after V1), post PS (2 weeks after V2), V13M (3 months after V1), pre B1 (6 months after V1), post B1 (2 weeks after V3), V33M (3 months after V3), pre B2 (6 months after V3), and post B2 (2 weeks after V4). V1-V4 indicate the first, second, third, and fourth dose vaccination, respectively. B1 and B2 indicate the first and second vaccine booster dose, respectively. PS, primary series.

Peripheral blood samples were serially drawn from patients nine times, including immediately before vaccination (baseline), 2 weeks after V1 (V12W), 2 weeks after V2 (after primary series; post PS), 3 months after V1 (V13M), 6 months after V1 (before the first vaccine booster dose; pre B1), 2 weeks after V3 (after the first vaccine booster dose; post B1), 3 months after V3 (V33M), 6 months after V3 (before the second vaccine booster dose; pre B2), and 2 weeks after V4 (after the second vaccine booster dose; post B2) (Figure 1).

### 2.3. Data Collection and Analysis

Serum and peripheral blood mononuclear cells (PBMCs) were obtained by centrifugation for 15 min at 1500× *g* at room temperature, aliquoted, and stored at −80 °C until use. Immunogenicity was measured as IgG against the SARS-CoV-2 spike receptor-binding domain (RBD) using an enzyme-linked immunosorbent assay kit (FUJIFILM Wako Pure Chemical Corporation, Tokyo, Japan) [23]. All procedures were conducted according to the manufacturer’s guidelines. The numbers of cells releasing interferon-γ (IFN-γ) in response to stimulation by SARS-CoV-2 peptides were measured with an enzyme-linked immunospot assay (ELISpot) using ELISpot Path: SARS-CoV-2 (S1scan+SNMO) Human IFN-γ and ELISpot Path: SARS-CoV-2 (Omicron BA.1, S1 scan) Human IFN-γ kits (Mabtech AB, Stockholm, Sweden), in accordance with the manufacturer’s instructions [24]. Briefly, the wells of a microplate precoated with the anti-IFN-γ monoclonal antibody (mAb1-D1K) were washed four times with phosphate-buffered saline (Nacalai Tesque, Kyoto, Japan) and then blocked with RPMI-1640 culture medium (Sigma Aldrich, St. Louis, MO, USA) containing 10% batch-tested fetal bovine serum (Sigma Aldrich, St. Louis, MO, USA). Cryopreserved PBMCs from whole blood samples were thawed, and 4 × 10^4^ PBMCs were seeded per well and stimulated with the SARS-CoV-2 S1 domain of the spike protein scanning peptide pool (Mabtech AB, Stockholm, Sweden) at a concentration of 2 μg/mL of each peptide for 14 h at 37 °C and 5% CO_2_. Negative (PBMCs treated with the peptide vehicle dimethyl sulfoxide) and positive (PBMCs stimulated with monoclonal anti-CD3 antibody [CD3-2]) controls were also included. After washing, the cells were removed, and IFN-γ production was assessed using a biotinylated anti-human IFN-γ detection antibody (1:2000, clone 7-B6-1), followed by incubation with streptavidin-alkaline phosphatase and 5-bromo-4-chloro-3’-indolyphosphate p-toluidine salt/nitro-blue tetrazolium chloride-plus substrate. Spots were counted using an automated spot analyzer (Zellnet Consulting Inc., Fort Lee, NJ, USA). The mean spot counts for the negative control wells were subtracted from the mean of the test wells to generate normalized readings, which were expressed as spot-forming units (SFU)/10^4^ PBMCs.

The serum creatinine-based estimated glomerular filtration rate (eGFR) was estimated using the Modification of Diet in Renal Disease Study equation, as recommended by the Japan Chronic Kidney Disease Initiative [25]. The 2D echocardiography was conducted by experts who were unaware of the study details, and the left ventricular ejection fraction was calculated using the modified Simpson’s rule.

### 2.4. Definitions

Coronary artery disease was defined as documented previous myocardial infarction, prior coronary revascularization, chest pain with myocardial ischemia detected by noninvasive tests, or >50% coronary stenosis, as demonstrated by angiography. Hypertensive heart disease included a history of hypertension and left ventricular hypertrophy in the absence of other causes. Aortic dissection was diagnosed based on imaging findings such as computed tomography. Hypertension was defined as systolic blood pressure of ≥140 mmHg, diastolic blood pressure of ≥90 mmHg, or a history of antihypertensive therapy. Dyslipidemia was defined as a total cholesterol level of ≥220 mg/dL or a history of receiving lipid-lowering therapy. Diabetes was defined as having a history of or a current diagnosis of diabetes mellitus, a fasting plasma glucose level of ≥126 mg/dL, a hemoglobin A1c value of ≥6.5%, or signs of diabetic retinopathy. Allergic rhinitis, hay fever, urticaria, and/or bronchial asthma were defined as allergic diseases. Patients with an eGFR < 60 mL/min/1.73 m^2^ were identified as having chronic kidney disease (CKD).

### 2.5. Statistical Analysis

All statistical analyses were conducted using the R software (version 4.0.3; R Foundation for Statistical Computing, Vienna, Austria). Normally distributed variables are expressed as mean values ± standard deviations, whereas non-parametric data are presented as medians and interquartile ranges. The Mann–Whitney U test was used to analyze non-normally distributed data. The paired Wilcoxon signed-rank test was used to compare the paired non-parametric data. Spearman’s rank correlation analysis was used to assess the correlation between variables. Statistical significance was set at two-sided *p* < 0.05.

## 3. Results

### 3.1. Baseline Characteristics of Study Participants

The demographic and clinical characteristics of the participants are presented in Table 1. A total of 26 patients with CVD (21 males; mean age, 72 years) were recruited for the present study. Coronary artery disease was the predominant diagnosis (20 patients), followed by hypertensive heart disease (4 patients). In total, 15 patients (58%) were diagnosed with CKD. The interval between vaccination and serum sampling was comparable. Of the 26 patients, 16 patients (62%) received BNT162b2 for their first to fourth doses, and 10 patients received BNT162b2 for their first and second doses and either BNT162b2 or mRNA-1273 for their third and fourth doses.

### 3.2. Dynamics of RBD-IgG Titers

**Figure 2:**
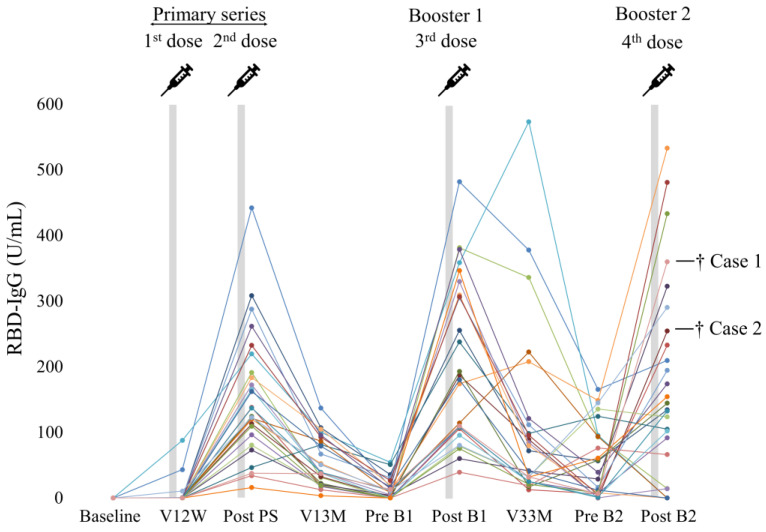
Dynamics of RBD-IgG titers. Serial changes in the RBD-IgG titers from the first vaccine administration. The RBD-IgG titers were evaluated at nine timepoints: including baseline (just before vaccination), V12W (2 weeks after V1), post PS (2 weeks after V2), V13M (3 months after V1), pre B1 (6 months after V1), post B1 (2 weeks after V3), V33M (3 months after V3), pre B2 (6 months after V3), and post B2 (2 weeks after V4). Each dot represents the antibody level of an individual patient. Lines connect data points obtained longitudinally from the same patient. V1-V4 indicate the first, second, third, and fourth dose vaccination, respectively. B1 and B2 indicate the first and second vaccine booster dose, respectively. Cases 1 and 2 represented patients with breakthrough infection. RBD, receptor-binding domain; PS, primary series.

**Figure 3:**
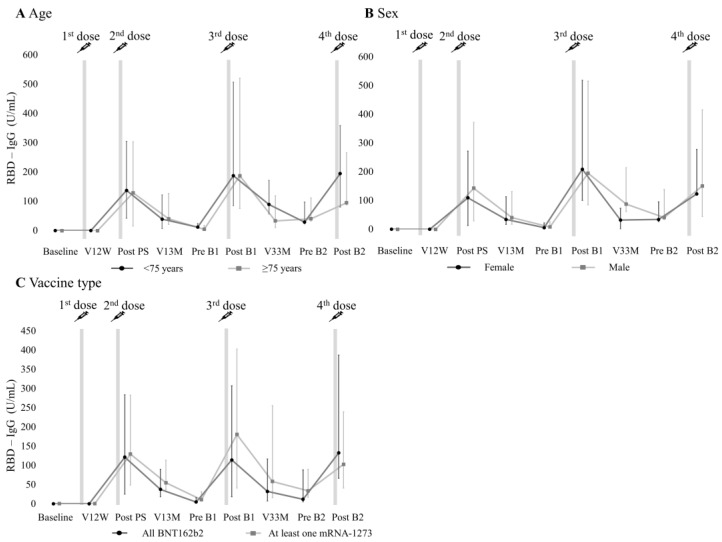
Trend in RBD-IgG titers according to age, gender, and vaccine type. The line plots show the median (interquartile range) of the RBD-IgG titers for (**A**) the different age groups [under (n = 15), over (n = 11) 75 years], for (**B**) gender [females (n = 5), males (n = 21)], and for (**C**) vaccine type [all BNT162b2 (n = 16), at least one mRNA-1273 (n = 10)]. RBD, receptor-binding domain.

**Figure 4:**
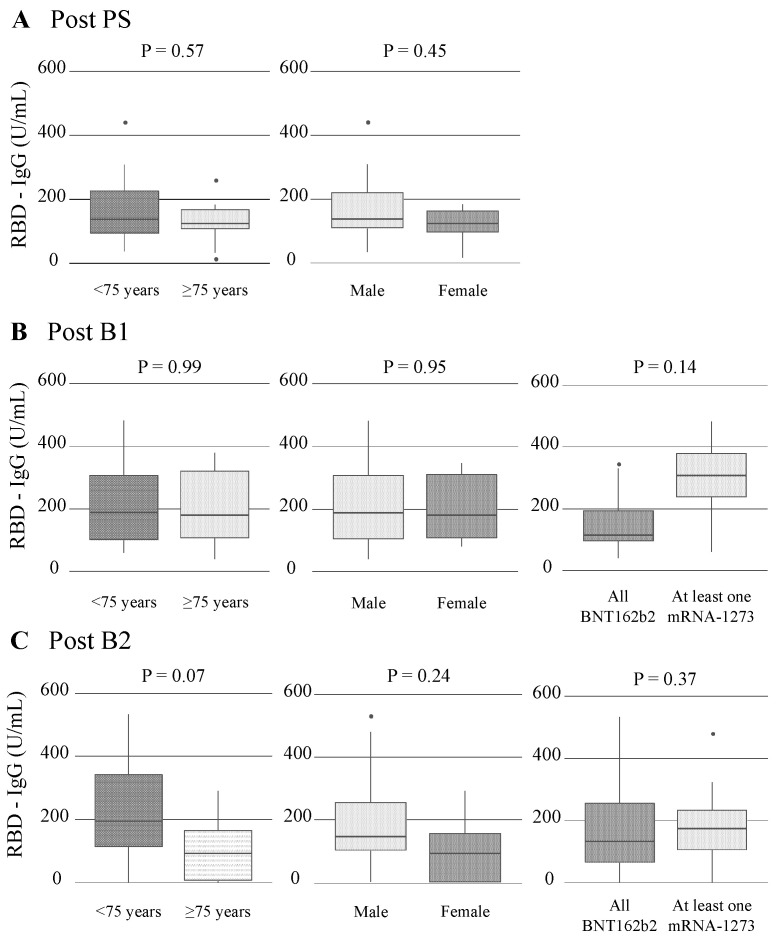
RBD-IgG titers after the primary and booster dose according to age, gender, and vaccine type. Each box indicates the interquartile range (top, the third quartile; bottom, the first quartile), with a horizontal line indicating the median, post (**A**) PS, (**B**) B1, and (**C**) B2. The Mann–Whitney U test was used to compare RBD-IgG titers between two groups. B1 and B2 indicate the first and second vaccine booster dose, respectively. RBD, receptor-binding domain; PS, primary series.

The serial changes in RBD-IgG titers of individual patients after the first vaccine dose are shown in Figure 2 and Table S1. RBD-IgG titers showed an elevation at post PS, followed by a decrease until pre B1. The levels peaked at post B1 and gradually declined until pre B2, followed by an increase again at post B2. This trend was observed regardless of age, sex, or vaccine type (Figure 3 and Table S1). There were no significant differences in RBD-IgG titers after the primary series and booster vaccinations based on age, sex, or vaccine type (Figure 4).

### 3.3. Vaccine Immunogenicity before and after Booster Vaccinations

#### 3.3.1. Humoral Immunogenicity

**Figure 5:**
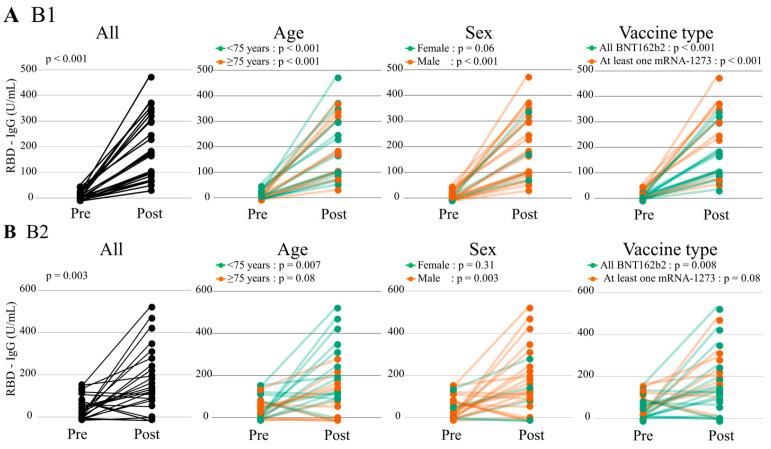
Humoral response before and after the booster dose. The RBD-IgG titers changes before and after (**A**) B1 and (**B**) B2. The paired Wilcoxon signed-rank test was used to compare RBD-IgG titers before and after the booster dose. B1 and B2 indicate the first and second vaccine booster dose, respectively. RBD, receptor-binding domain.

The RBD-IgG titers at post B1 and B2 (183.7 [106.2–308.8] and 139.5 [72.6–249.1]) U/mL) significantly increased compared with those at pre B1 and B2 (8.5 [0.0–17.2] and 34.0 [6.8–93.5] U/mL, paired Wilcoxon signed-rank test, *p* < 0.001 and *p* = 0.003, respectively) (Figure 5 and Table S1). Similar findings were observed in patients aged < 75 years, males, and those vaccinated with BNT162b2 only. Among patients aged ≥ 75 years and those vaccinated with at least one mRNA-1273, the RBD-IgG titers at post B1 only significantly increased compared with those at pre B1.

#### 3.3.2. Cellular Immunity

**Figure 6:**
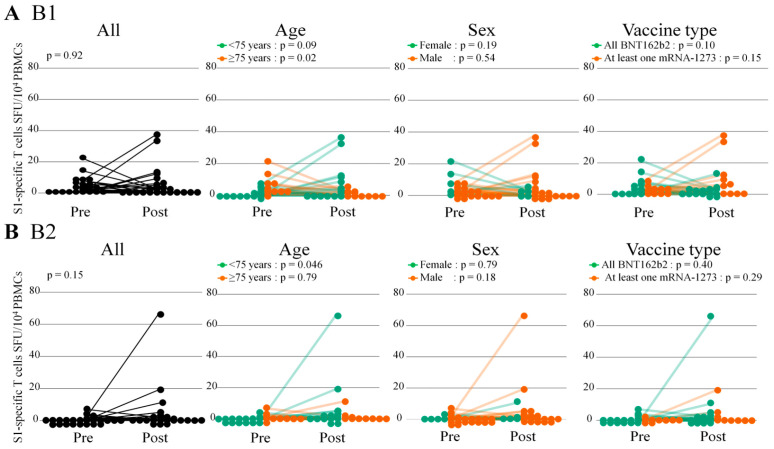
Cellular response against S1 before and after the booster dose. Cellular response after the booster dose by INF-γ secreting T cells. Assessed by ELISpot after stimulation by SARS-CoV-2 (S1scan+SNMO) Human IFN-γ kits and expressed as SFU/10^4^ PBMCs. The paired Wilcoxon signed-rank test was used to compare the S1-specific T cell responses before and after the booster dose. (**A**,**B**) B1 and B2 indicate the first and second vaccine booster dose, respectively. INF-γ, interferon-γ; SFC, spot-forming units; PBMCs, peripheral blood mononuclear cells; ELISpot, Enzyme-Linked immunoSPOT.

**Figure 7:**
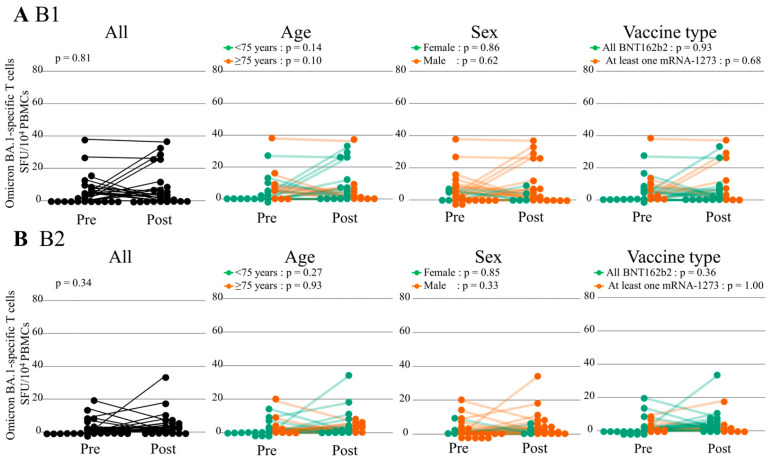
Cellular response against Omicron BA.1 before and after the booster dose. Cellular response after the booster dose by INF-γ secreting T cells. Assessed by ELISpot after stimulation by SARS-CoV-2 (Omicron BA.1, S1 scan) Human IFN-γ kits and expressed as SFU/10^4^ PBMCs. The paired Wilcoxon signed-rank test was used to compare the Omicron BA.1-specific T cell responses before and after the booster dose. (**A**,**B**) B1 and B2 indicate the first and second vaccine booster dose, respectively. INF-γ, interferon-γ; SFC, spot-forming units; PBMCs, peripheral blood mononuclear cells; ELISpot, Enzyme-Linked immunoSPOT.

The S1-specific IFN-γ-releasing T cell responses before and after the first and second booster doses overall remained stable (Figure 6 and Table S2). Among the subgroup of patients aged < 75 years, the S1-specific T cell responses at post B2 significantly increased compared with those at pre B2 (paired Wilcoxon signed-rank test, *p* = 0.046) and tended to increase before and after B1 (*p* = 0.09). There were no significant differences in S1-specific T cell responses before and after the booster dose regardless of sex and vaccine type. However, the specific T cell activation responses against Omicron BA.1 after booster doses were poor regardless of age, sex, and vaccine type (Figure 7 and Table S3).

### 3.4. Correlation of RBD-IgG Titers and Numbers of Specific T Cells

**Figure 8:**
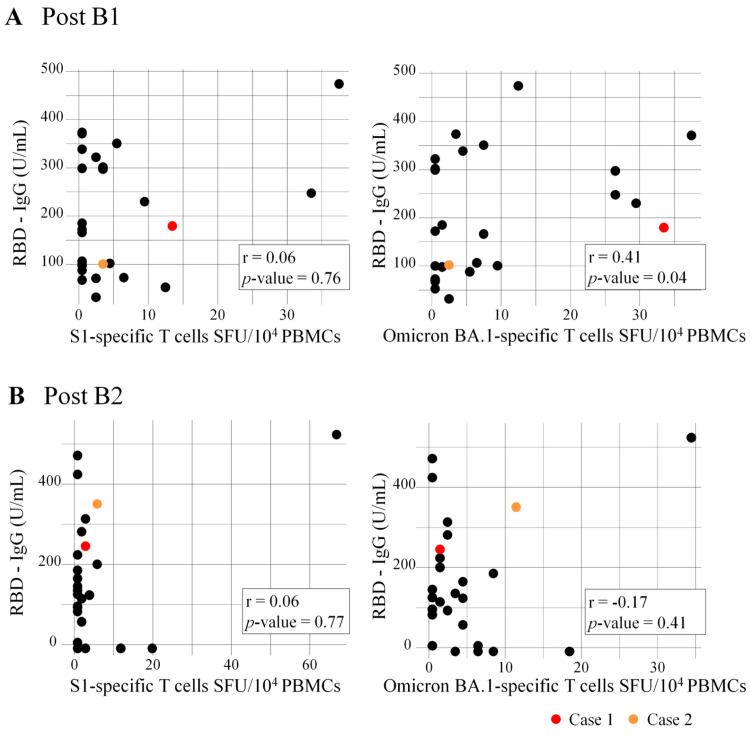
Correlation between RBD-IgG titers and numbers of specific T cells at post (**A**) B1 and (**B**) B2. Spearman’s rank correlation analysis was used to assess the correlation between variables. B1 and B2 indicate the first and second vaccine booster dose, respectively. Cases 1 and 2 represented patients with breakthrough infection. RBD, receptor-binding domain; SFC, spot-forming units; PBMCs, peripheral blood mononuclear cells.

The RBD-IgG titers at post B1 showed positive correlation with the numbers of Omicron BA.1-specific T cells (r = 0.41, *p* = 0.04), but not with the numbers of S1-specific T cells (r = 0.06, *p* = 0.76) (Figure 8). There was no significant correlation between the RBD-IgG titers and the numbers of S1- and Omicron BA.1-specific T cells at post B2 (r = 0.06, *p* = 0.77 and r = −0.17, *p* = 0.41, respectively).

### 3.5. Safety and Breakthrough Infection

No severe local or systemic adverse events occurred after the vaccination during the study period. In this study, two patients who had high antibody titers 2 weeks after the second booster vaccination (Cases 1 and 2: 254.5 and 360.0 U/mL, respectively) had breakthrough infection (Figure 2 and Figure 8). They developed COVID-19 around 4 and 11 months after B2, respectively, but no deaths or hospitalizations were reported.

## 4. Discussion

In the present study, we investigated the longitudinal humoral and cellular responses to primary series and booster doses of the mRNA COVID-19 vaccine in naïve patients with CVD. The three main findings of this study were as follows: (i) the RBD-IgG titers increased 2 weeks after the primary series and booster doses and waned by 6 months after vaccination, (ii) the S1-specific T cell responses in patients aged < 75 years were favorable before and after booster doses, and (iii) the Omicron BA.1-specific T cell responses were poor.

We previously reported that the antibody titers in patients with CVD increased 2 weeks after the primary series of mRNA COVID-19 vaccines, which were significantly lower than those in healthcare workers (HCWs) [26]. However, few studies have examined the long-term humoral response after primary series in patients with CVD. Thus, we investigated the longitudinal humoral response and clarified, for the first time, the dynamics of RBD-IgG titers after the primary series and booster doses. The RBD-IgG titers were elevated after the primary series and rapidly declined within 6 months. This is in line with the previously reported kinetics of antibodies in HCWs [19,27,28] and patients undergoing hemodialysis [29,30]. The RBD-IgG titers after the first and second booster doses in patients with CVD significantly increased and waned after 6 months. The kinetics of the antibody response to the booster doses were consistent with those reported in previous studies [31]. These data underscore the need to consider booster doses in patients with CVD in order to maintain a robust antibody response over time.

The cellular immune response against SARS-CoV-2 has been assessed in healthy adults [32,33], individuals with convalescent COVID-19 [34], and patients with immune-mediated inflammatory diseases [35]. However, little evidence is available on the impact of booster-dose vaccine-induced T cell responses in patients with CVD. We demonstrated for the first time that vaccine boosting increases the frequency of S1-specific IFNγ-releasing T cells in patients under 75 years of age. Previous studies have shown that older age is associated with a weakened cellular immune response to COVID-19 vaccines [36,37,38]. Additional factors that likely affect the poor vaccine response to COVID-19 include comorbid conditions with age [39]. In our study, 77%, 31%, and 19% of the patients had hypertension, diabetes, and previous myocardial infarction, respectively, which may have contributed to impaired cellular immune responses in patients with CVD. Our data also showed that the specific T cell activation responses against Omicron BA.1 remained stable before and after the vaccine booster. In Japan, the fourth dose (second booster) of the ancestral-strain monovalent COVID-19 mRNA vaccine was rolled out for individuals aged ≥ 60 years and those aged 18–59 years with underlying health conditions, such as chronic heart disease. The vaccine type may have affected the poor specific T cell activation responses against Omicron BA.1. Both the S1- and Omicron BA.1-specific IFN-γ-releasing T cell responses were markedly impaired in patients aged ≥ 75 years, suggesting that older patients are vulnerable and need to be prioritized for Omicron-adapted mRNA vaccines as a booster dose. In this study, two patients had breakthrough infections with high antibody titers 2 weeks after the second booster vaccination. Therefore, the continuation of appropriate infection prevention practices and an additional booster dose may be necessary, especially to protect older patients with CVD, regardless of the humoral response.

It has been observed that patients with cardiovascular disease are at an increased risk of developing severe complications due to SARS-CoV-2 infection [1,2,3,4,5,6,7,8,9]. Therefore, more aggressive vaccinations may be recommended for these patients. However, multiple reports have arisen about cardiovascular complications following the mRNA COVID-19 vaccine. In a systematic review, thrombosis was frequently reported, followed by stroke, myocarditis, myocardial infarction, pulmonary embolism, and arrhythmia [40]. Furthermore, thrombosis was common in the BNT162b2 cohort, while stroke was common with mRNA-1273. Myocarditis has been recognized as a rare complication of the mRNA COVID-19 vaccine, especially in young adult and adolescent males [41,42]. We should discuss the balance of the risk of complications with vaccination versus cardiac and other risks from COVID-19 infection and then decide on vaccination depending on the patient’s condition.

Our study had some limitations. First, the sample size is not enough. However, we are not aware of other similar studies that have collected longitudinal data over 1 year and 9 months from patients with CVD. Second, because we excluded patients with a previous diagnosis of COVID-19, some patients may have had unrecognized infection-induced immunity. Third, the proportion of women was small (n = 5) and the numbers of male and female patients are uneven; therefore, we were unable to analyze the effect of sex on humoral and cellular responses induced by the primary and booster doses of the mRNA COVID-19 vaccine. Fourth, age is not normally distributed, so data might be biased. Finally, because this study enrolled only Japanese patients with CVD, whether our findings can be extrapolated to a non-Japanese population remains uncertain.

## 5. Conclusions

Our study showed that humoral immunity in patients with CVD was strong immediately after receiving the mRNA-based COVID-19 vaccine in primary and booster doses but gradually weakened over several months. The S1-specific T cell responses in patients aged < 75 years were favorable before and after the booster doses, whereas cellular immunity to the Omicron variant was impaired. These results suggest that booster doses are effective in maintaining long-term antibody levels and have implications for vaccination strategies in patients with CVD moving forward. Furthermore, additional booster doses, including Omicron variant-adapted mRNA vaccines, may be recommended for patients with CVD, regardless of age.

## Figures and Tables

**Table 1:** Baseline characteristics of the study participants.

	Subjects (N = 26)
Age, y	72 ± 7
Male	21 (81%)
Hypertension	20 (77%)
Dyslipidemia	18 (69%)
Diabetes	8 (31%)
Allergic disease	4 (15%)
Systolic blood pressure, mmHg	131 ± 14
Heart rate, beats per minutes	71 ± 12
Diagnosis	
Coronary artery disease	20 (77%)
Hypertensive heart disease	4 (15%)
Dilated cardiomyopathy	1 (4%)
Aortic dissection	1 (4%)
Previous myocardial infarction	5 (19%)
Previous coronary revascularization	12 (46%)
Paroxysmal or persistent atrial fibrillation	2 (8%)
Laboratory data	
White blood cell count, ×10^3^/μL	5.9 ± 1.7
Hemoglobin, g/dL	14.0 ± 1.4
Platelet count, ×10^4^/μL	19.6 ± 4.1
Creatinine-based estimated glomerular filtration rate, mL/min/1.73 m^2^	58.0 ± 14.4
Chronic kidney disease	15 (58%)
Low-density lipoprotein-cholesterol, mg/dL	91.5 ± 21.8
Hemoglobin A_1c_, %	6.3 ± 0.9
N-terminal pro-B-type natriuretic peptide, pg/mL	116.5 (48.0–356.0)
High-sensitivity Troponin I, pg/mL	3.70 (2.10–12.0)
Left ventricular ejection fraction, %	54 ± 10
Medications	
Renin–angiotensin–aldosterone system inhibitors	11 (42%)
Beta-blockers	10 (38%)
Diuretics	3 (12%)
Statins	14 (54%)
Antiplatelet drugs	12 (46%)
Anticoagulant drugs	4 (15%)
Intervals between, day	
V1 and sampling (V12W)	14.7 ± 2.4
V2 and sampling (post PS)	15.2 ± 2.1
B1 and sampling (post B1)	15.3 ± 2.6
B2 and sampling (post B2)	14.9 ± 2.9
V1 and V2	21.6 ± 1.9
V2 and B1	222.7 ± 16.8
B1 and B2	188.0 ± 9.9
Vaccine type	
V1	
BNT162b2	26 (100%)
V2	
BNT162b2	26 (100%)
V3	
BNT162b2	19 (73%)
mRNA-1273	7 (27%)
V4	
BNT162b2	18 (69%)
mRNA-1273	8 (31%)

Values are reported as mean ± SD or median (25th–75th percentile) for quantitative variables and as n (%) for categorical variables. V12W, 2 weeks after V1; PS, primary series; B1, first vaccine booster dose; B2, second vaccine booster dose, V1, first vaccination; V2, second vaccination; V3, third vaccination; V4, fourth vaccination.

## Data Availability

Data presented in this study are available upon request from the corresponding author. The data are not publicly available due to privacy or ethical reasons.

## Supplementary Materials

The following supporting information can be downloaded at: https://www.mdpi.com/article/10.3390/vaccines12070786/s1, Table S1: RBD-IgG titers (U/mL); Table S2: S1-specific T cell response (SFU/10^4^ PBMCs); Table S3: Omicron BA.1-specific T cell response (SFU/10^4^ PBMCs).
