# Supplementary material for: Humoral and Cellular Response Induced by Primary Series and Booster Doses of mRNA Coronavirus Disease 2019 Vaccine in Patients with Cardiovascular Disease: A Longitudinal Study
Source: Vaccines (Basel). 2024 Jul 17;12(7):786. doi: 10.3390/vaccines12070786 (PMC11281625; doi:10.3390/vaccines12070786)
Supplement: Supplementary file 1 [file vaccines-12-00786-s001.zip › vaccines-3059345-supplementary.pdf]

Supplemental Table S1 RBD-IgG titers (U/mL)

	**Overall** (N = 26)	**Age ≤75** (N = 15)	**Age >75** (N = 11)	**Female** (N = 5)	**Male** (N = 21)	**All BNT162b2** (N = 16)	**At least one mRNA-1273** (N = 10)
Baseline	0.0 [0.0 - 0.0]	0.0 [0.0 - 0.0]	0.0 [0.0 - 0.0]	0.0 [0.0 - 0.0]	0.0 [0.0 - 0.0]	NA	NA
V12W	0.0 [0.0 - 0.0]	0.0 [0.0 - 0.0]	0.0 [0.0 - 0.0]	0.0 [0.0 - 0.0]	0.0 [0.0 - 0.0]	NA	NA
Post PS	130.8 [99.5 - 189.4]	137.2 [95.1 - 226.1]	124.3 [108.9 - 167.3]	122.8 [96.2 - 162.3]	137.2 [109.5 - 219.6]	NA	NA
V13M	39.1 [21.0 - 85.2]	39.1 [32.0 - 86.8]	39.0 [18.1 - 82.8]	50.6 [17.5 - 79.0]	39.0 [22.3 - 86.6]	NA	NA
Pre B1	8.5 [0.0 - 17.2]	11.7 [1.1 - 28.7]	4.9 [0.0 - 12.6]	10.7 [0.1 - 16.9]	7.7 [0.0 - 17.3]	NA	NA
Post B1	183.7 [106.2 - 308.8]	187.3 [101.7 - 306.1]	180.1 [106.9 - 319.6]	180.1 [108.1 - 309.5]	187.3 [105.7 - 306.9]	144.1 [104.8 - 221.0]	281.2 [138.7 - 373.7]
V33M	56.7 [27.0 - 108.3]	89.2 [32.5 - 159.7]	32.3 [22.0 - 82.0]	32.3 [31.8 - 40.6]	84.3 [25.4 - 121.2]	32.5 [25.1 - 85.5]	97.0 [49.1 - 282.3]
Pre B2	34.0 [6.8 - 93.5]	28.8 [5.7 - 110.0]	39.2 [7.6 - 68.5]	12.0 [7.6 - 60.8]	39.2 [6.5 - 93.6]	10.0 [5.6 - 64.6]	74.9 [31.4 - 117.3]
Post B2	139.5 [72.6 - 249.1]	194.6 [114.3 - 341.3]	91.7 [7.1 - 164.3]	91.7 [0.0 - 154.5]	144.6 [102.2 - 254.5]	128.1 [53.2 - 209.6]	191.8 [112.3 - 276.1]

Data are expressed as median (25th–75th percentile).
RBD, receptor-binding domain; V12W, 2 weeks after V1; PS, primary series; B1, first vaccine booster dose; V13M, 3 months after V1; V33M, 3 months after V3; B2, second vaccine booster dose, V1, first vaccination; V2, second vaccination; V3, third vaccination; V4, fourth vaccination. NA, not applicable.

Supplemental Table S2 S1- specific T-cells response (SFU/10^4 PBMCs)

	**Overall** (N = 26)	**Age ≤75** (N = 15)	**Age >75** (N = 11)	**Female** (N = 5)	**Male** (N = 21)	**All BNT162b2** (N = 16)	**At least one mRNA-1273** (N = 10)
Pre B1	2.5 [0.0 - 5.0]	0.0 [0.0 - 2.5]	3.0 [3.0 - 8.0]	8.0 [3.0 - 14.0]	2.0 [0.0 - 3.0]	2.5 [0.0 - 6.5]	2.5 [0.0 - 3.0]
Post B1	2.0 [0.0 - 4.8]	3.0 [0.0 - 10.5]	0.0 [0.0 - 2.5]	3.0 [0.0 - 3.0]	2.0 [0.0 - 5.0]	1.0 [0.0 - 3.0]	5.5 [0.0 - 11.2]
Pre B2	0.0 [0.0 - 0.0]	0.0 [0.0 - 0.0]	0.0 [0.0 - 1.5]	0.0 [0.0 - 0.0]	0.0 [0.0 - 0.0]	0.0 [0.0 - 3.0]	0.0 [0.0 - 0.0]
Post B2	0.0 [0.0 - 2.0]	1.0 [0.0 - 4.0]	0.0 [0.0 - 1.0]	0.0 [0.0 - 1.0]	0.0 [0.0 - 2.0]	0.5 [0.0 - 2.2]	0.0 [0.0 - 1.8]

Data are expressed as median (25th–75th percentile).
SFU, spot-forming units; PBMCs, peripheral blood mononuclear cells; B1, first vaccine booster dose; B2, second vaccine booster dose.

Supplemental Table S3 Omicron BA.1- specific T cells response (SFU/10^4 PBMCs)

	**Overall** (N = 26)	**Age ≤75** (N = 15)	**Age >75** (N = 11)	**Female** (N = 5)	**Male** (N = 21)	**All BNT162b2** (N = 16)	**At least one mRNA-1273** (N = 10)
Pre B1	3 [0 - 8]	0 [0 - 4]	6 [2 - 8]	5 [0 - 6]	2 [0 - 9]	2 [0 - 6]	4 [1 - 10]
Pre B2	2 [0 - 8]	5 [0 - 19]	1 [0 - 5]	0 [0 - 4]	3 [0 - 12]	2 [0 - 6]	5 [0 - 22]
Pre B3	0.0 [0.0 - 3.0]	0.0 [0.0 - 2.5]	1.0 [0.0 - 3.5]	1.0 [0.0 - 2.0]	0.0 [0.0 - 3.0]	0.0 [0.0 - 2.2]	1.5 [0.3 - 3.8]
Post B3	2.0 [0.3 - 5.5]	1.0 [0.0 - 6.0]	3.0 [1.5 - 5.0]	2.0 [0.0 - 3.0]	2.0 [1.0 - 6.0]	3.5 [1.0 - 6.5]	1.0 [0.0 - 2.0]

Data are expressed as median (25th–75th percentile).
SFU, spot-forming units; PBMCs, peripheral blood mononuclear cells; B1, first vaccine booster dose; B2, second vaccine booster dose.
